# The relationship between physical exercise and social-emotional competence in college students: the chain mediation of peer relationships and self-efficacy

**DOI:** 10.3389/fpsyg.2026.1836284

**Published:** 2026-07-28

**Authors:** Hongbo Zhao, Jialu Zhang

**Affiliations:** Liaoning Normal University, Dalian City, Liaoning Province, China

**Keywords:** college students, peer relationships, physical exercise, self-efficacy, social-emotional competence

## Abstract

**Introduction:**

College students face prominent emotional and interpersonal challenges under current social and academic pressures, and social-emotional competence is essential for their mental health and healthy development. This study investigates the relationship between physical exercise and social-emotional competence among college students, as well as the mediating roles of, peer relationships, and self-efficacy in this relationship.

**Methods:**

A convenience sample of 517 college students from several universities in Liaoning Province was surveyed. Data were collected using the Physical Exercise Scale, Peer Relationship Scale, Self-Efficacy Scale, and Social-Emotional Competence Scale. Data were analyzed using SPSS 26.0, the PROCESS 3.4 plugin, and the Bootstrap method to test the chained mediation effects of peer relationships and self-efficacy.

**Results:**

(1) Physical exercise, peer relationships, and self-efficacy were all positively correlated with college students' social-emotional competence. (2) Physical exercise was positively correlated with college students' peer relationships, self-efficacy, and social-emotional competence; peer relationships were positively correlated with self-efficacy and social-emotional competence; and self-efficacy was positively correlated with social-emotional competence.

**Discussion:**

This research constructs a chained mediation model, systematically clarifying the relationship between physical exercise and social-emotional competence among college students, as well as the mediating roles of, peer relationships, and self-efficacy in this relationship. The research outcomes offer solid theoretical references and feasible practical enlightenment for carrying out college mental health education and optimizing students' social-emotional ability training programs.

## Introduction

Against the backdrop of high-quality development in higher education, college students' mental health and social adaptation abilities have become critical issues requiring urgent attention (Zhang and Liu, 2021). Compared to elementary school students, whose minds are not yet fully mature, and middle-aged and older adults, whose psychological traits tend to be stable, college students are at a transitional stage between campus life and society, and their psychological characteristics hold unique research value. Today, multiple pressures—including an accelerating pace of life, intensifying academic competition, and increasing uncertainty in the job market—have led to increasingly prominent issues among contemporary college students, such as emotional dysregulation, interpersonal alienation, and social anxiety. This phenomenon is particularly pronounced in the post-pandemic context (Balayar et al., 2025). Social-emotional competence, as a core competency enabling individuals to cope with social challenges, regulate their emotions, and build healthy interpersonal relationships, is not only a crucial pillar of college students' mental health but also a key factor directly influencing their academic development, career planning, and lifelong well-being (Sun et al., 2025). Research indicates that social-emotional competence helps students improve their academic performance (Zhiying et al., 2024), enhance their sense of well-being (Tian et al., 2021), and promote mental health (Godara et al., 2021). Therefore, effectively fostering college students' social-emotional competencies has become an urgent need in higher education institutions' mental health education and talent development efforts (Cook, 2022). Physical exercise, as a low-cost, high-return intervention, may have a positive association with social-emotional competencies. Relevant studies indicate that participating in physical exercise can significantly improve college students' mental health, effectively enhance subjective well-being, alleviate negative emotions such as anxiety and depression, and simultaneously optimize their social adaptation (Liu et al., 2025). According to the theory of emotional socialization, an individual's social-emotional competence is not innate but is gradually acquired and developed through social interaction and accumulated experience (Spinrad and Eisenberg, 2019). In a college setting, physical exercise—particularly team sports—is not merely a form of physical exercise but also helps establish positive peer relationships (Weiss and Duncan, 1992); strong peer relationships often provide individuals with positive feedback, further reinforcing their sense of self-efficacy (Bao, 2025). A higher sense of self-efficacy enhances an individual's ability to participate in social activities and regulate negative emotions, thereby promoting social-emotional competence (Liu et al., 2024a). Currently, the academic community has conducted some research on physical exercise, peer relationships, self-efficacy, and social-emotional competence; however, most studies have only examined direct associations between two or three variables—such as physical exercise and self-efficacy (Wu et al., 2022) or peer relationships and physical exercise (Pan et al., 2021)—and the research subjects have often been limited to adolescents (Yan and Lu, 2025). Very few studies have incorporated all four variables—physical exercise, peer relationships, self-efficacy, and social-emotional competence—into a single research framework to examine the chain mediation pathway between peer relationships and self-efficacy. Based on this, the present study aims to thoroughly analyze the relationship between physical exercise and social-emotional competence among college students. At the same time, it innovatively incorporates peer relationships and self-efficacy as chained mediating variables to construct a “physical exercise–peer relationships–self-efficacy–social-emotional competence” chained mediation model, thereby providing new theoretical foundations and practical directions for mental health education and interventions in higher education institutions.

### The relationship between physical exercise and social-emotional competence among college students

Social-emotional competence (SEC), as a core competency for individual social adaptation, has garnered increasing attention. It refers to an individual's comprehensive ability to identify and manage emotions, establish positive interpersonal relationships, and make responsible decisions in social interactions, and serves as an important protective factor for college students' mental health (Chernyshenko et al., 2018). Research by CASEL (Collaborative for Academic, Social, and Emotional Learning) indicates that social-emotional competence is not innate but rather malleable; it is a non-cognitive ability distinct from traditional intellectual factors (Wang et al., 2019). Physical exercise refers to physical activities that combine natural forces with health measures, with the aim of enhancing physical fitness, regulating physical and mental well-being, and enriching cultural life (Hong Kong Sports Institute, 2000). Social interaction theory suggests that physical exercise—especially group physical exercise—provides individuals with opportunities to interact with others. Through this process, individuals can learn to effectively manage interpersonal relationships, resolve conflicts, and demonstrate support and empathy, among other social skills (Weiss and Wiese-Bjornstal, 2009). Existing research indicates that physical exercise is associated with social-emotional competencies in college students (Liu et al., 2023). Students who engage in physical exercise over the long term, particularly those participating in team sports, demonstrate better social adaptation and interpersonal skills (Dong et al., 2023). Due to its unique social attributes, physical exercise possesses rich psychological dimensions such as emotional experiences and moral development, making it a valuable vehicle for cultivating social-emotional competencies (Wang and Bu, 2023). Based on this, this study proposes Hypothesis 1 (H1): Physical exercise is positively correlated with college students' social-emotional competencies.

### The mediating role of peer relationships

One of the mediating mechanisms explored in this study is the mediating role of peer relationships. In the process by which physical exercise influences an individual's psychological development, the external interpersonal environment serves as an indispensable mediating channel, and peer relationships—as the primary interpersonal relationships during the college years—play a mediating role (Shi et al., 2025). Peer relationships refer to interpersonal relationships characterized by shared activities and mutual collaboration among individuals at the same or similar stages of psychological development (Liu et al., 2024b). Interpersonal relationship theory posits that peer interactions directly contribute to the formation of social-emotional competencies, such as emotional regulation and social skills, and reflect the intrinsic connection between the external environment and individual psychological development. Research indicates that regular participation in physical exercise facilitates the establishment of peer relationships (Zhou et al., 2025). During physical exercise, mutual encouragement and collective achievements play an irreplaceable role in maintaining positive peer relationships (Yang et al., 2013). At the same time, the level of peer relationships is significantly positively correlated with college students' social-emotional competencies; the higher the quality of peer relationships, the higher the individual's level of social-emotional competencies (Lee and Ahn, 2025). In particular, positive peer relationships can effectively reduce stress and negative emotions in adolescents' academic and daily lives, thereby enhancing their social-emotional competencies (Yan and Li, 2021). Conversely, negative peer relationships may hinder the development of an individual's social-emotional competence (Dai and Peng, 2021) and increase the risk of various psychological problems (Zhong et al., 2017). Therefore, peer relationships play a role that cannot be overlooked in the relationship between physical exercise and college students' social-emotional competence. Based on this, this study proposes Hypothesis 2 (H2): Peer relationships mediate the relationship between physical exercise and college students' social-emotional competence.

### The mediating role of self-efficacy

Another mediating mechanism explored in this study is the mediating role of self-efficacy. Self-efficacy refers to an individual's assessment and judgment of their ability to perform a specific behavior (Bandura, 1986). According to Bandura's theory of self-efficacy, self-efficacy serves as an internal motivational mechanism for individual action and is positively correlated with emotional responses (Chahal et al., 2026). From a forward-path perspective, physical exercise is associated with self-efficacy. Participating in physical exercise not only enhances college students' physical fitness but also enables them to gain a sense of accomplishment and accumulate successful experiences while overcoming physiological limits and mastering motor skills, thereby fostering self-efficacy (Sheng et al., 2025). From a backward path analysis, self-efficacy is also associated with college students' social-emotional competencies. According to social cognitive theory, individuals with high self-efficacy tend to view difficulties as challenges; when facing complex social situations, they are able to mobilize more cognitive resources for emotional regulation and interpersonal interaction (Qiu et al., 2025). Therefore, compared to college students with low self-efficacy, those with high self-efficacy possess stronger social-emotional competencies. In other words, self-efficacy plays an indirect role in the relationship between physical exercise and college students' social-emotional competencies. Based on this, the present study proposes Hypothesis 3 (H3): Self-efficacy mediates the relationship between physical exercise and college students' social-emotional competencies.

### Chain mediation of peer relationships and self-efficacy

Research has shown that there is a positive correlation between peer relationships and self-efficacy (Bao, 2025). The mirror self-theory suggests that others' evaluations and feedback are important sources of an individual's self-perception (Katula et al., 1998). For college students, whose lives revolve primarily around campus life, peers are the people with whom they have the closest daily contact; therefore, their peers' attitudes and feedback serve as a reference point for forming their self-perception. As individuals construct their self-perception, they also gradually build confidence in their own abilities, leading to a sense of self-efficacy (Fu et al., 2026). In the context of physical exercise, positive peer relationships mean that individuals are more likely to receive encouragement, affirmation, and positive feedback from teammates (Chen et al., 2019). Such positive peer relationships can effectively reduce an individual's social anxiety, affirm their abilities, and foster a sense of self-efficacy (Hughes and Chen, 2011). This clearly illustrates that peer relationships and self-efficacy exist in a specific sequential relationship rather than in parallel. Furthermore, self-efficacy—generated through the internalization of peer support—serves as an intrinsic psychological motivator that prompts individuals to actively engage in social exploration, learn to see things from others' perspectives, and manage their own emotions. This process enables them to continuously refine core competencies such as emotional regulation and responsible decision-making, thereby enhancing their social-emotional competence (Zhang and Hasibagen, 2022). Therefore, peer relationships, as an environmental factor, are a key driver of self-efficacy, and together they constitute the underlying pathway linking physical exercise to social-emotional competence. Based on this, this study proposes Hypothesis 4 (H4): Peer relationships and self-efficacy play a chained mediating role in the relationship between physical exercise and college students' social-emotional competence.

**Figure 1:**
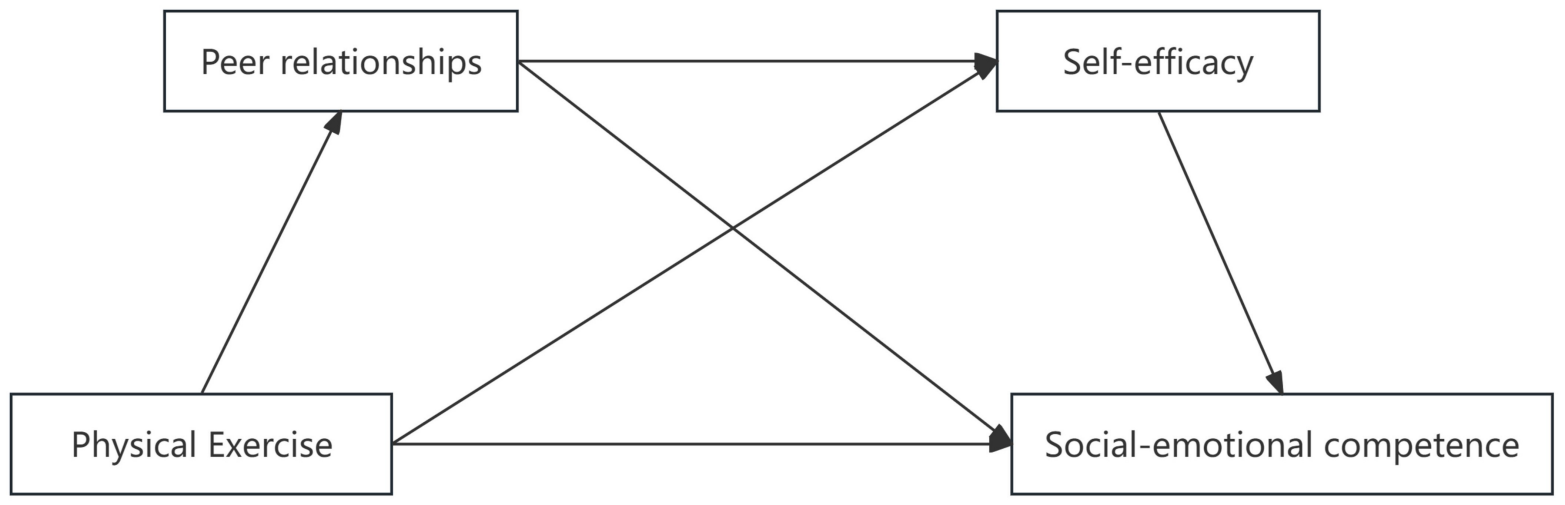
Hypothesis model.

Consequently, the research hypothesis model is constructed as shown in Figure 1.

### Study participants

This study employed convenience sampling to select a total of five institutions of different types—including comprehensive universities, science and engineering universities, and teacher training colleges—within Liaoning Province for the survey. The questionnaire was distributed online and covered five sections: basic information, level of physical exercise, social-emotional competencies, peer relationships, and self-efficacy. The purpose of the survey, the principle of voluntary participation, and other relevant matters were explained in detail in the instructions section of the electronic questionnaire. The participants' ages ranged primarily from 18 to 24, covering first-, second-, third-, and fourth-year undergraduate students. A total of 540 questionnaires were distributed. After collection, invalid questionnaires were screened through manual, item-by-item verification. Twenty-three invalid questionnaires were excluded based on four criteria: excessively short response times, repetitive or pattern-like answers, missing core items, and logical contradictions in responses. Ultimately, 517 valid questionnaires were obtained, resulting in a valid response rate of 95.74% and an overall missing data rate of 4.26%.Among the respondents, 264 were male and 253 were female; the number of students in the first, second, third, and fourth years of undergraduate study were 118, 132, 139, and 128, respectively. The basic characteristics of the survey participants are shown in Table 1.

**Table 1:** Basic demographic information of respondents.

Demographic variables	Category	Number	Percentage (%)
Gender	Male	264	51.0
	Female	253	48.9
Grade	Freshman	118	22.8
	Sophomore	132	25.5
	Junior	139	26.9
	Senior	128	24.8
Place of origin	City	344	66.5
	Rural	173	33.5
Whether an only child	Yes	129	25.0
	No	388	75.0
Total		517	100

Gender: 1, Male; 2, Female; Grade Level: 1, Freshman; 2, Sophomore; 3, Junior; 4, Senior; Place of Origin: 1, Urban; 2, Rural; Only Child: 1, Yes; 2, No.

## Research tools

### Physical Exercise Level Scale

The Physical Exercise Level Scale, revised by Liang Deqing et al. in 1994 (Liang, 1994), was used to assess the physical exercise levels of college students. This scale consists of three dimensions—exercise intensity (e.g., “How intense is your physical exercise?”), exercise duration (e.g., “How long do you exercise each time?”), and exercise frequency (e.g., “How many times a month do you exercise?”)—each comprising one item. A 5-point Likert scale was used, with exercise intensity and frequency scored on a scale of 1–5, and duration scored on a scale of 0–4. The total exercise volume is calculated as the product of exercise intensity, frequency, and duration; a higher score indicates a greater total exercise volume. In this study, the Cronbach's α coefficient for this scale was 0.777. The results of the construct validity analysis (single-factor structure, factor loadings 0.62–0.86, CR = 0.72, AVE = 0.51; acceptance criteria: factor loadings ≥ 0.50, CR ≥ 0.70, AVE ≥ 0.50).

### Social-Emotional Competence Scale

The Social-Emotional Competence Scale for College Students (Chen et al., 2023), developed by Chen Zhaojun and colleagues in 2023, was used to assess the social-emotional competence of college students. The scale consists of four dimensions—self-relationship competence (e.g., “I am aware of my strengths and weaknesses”), interpersonal competence (e.g., “I can establish good interpersonal relationships with others”), group-relationship competence (e.g., “I respect myself and also hope to be respected by others”), and responsible decision-making competence (e.g., “I can take responsibility for my own actions”)—and comprises a total of 26 items. The scale uses a 5-point Likert scale, with scores ranging from 1 to 5 to indicate responses from “strongly disagree” to “strongly agree.” After scoring each dimension separately, the scores for each item across the four dimensions are summed and divided by the number of items to obtain an average score; a higher score indicates a higher level of social-emotional competence. In this study, the Cronbach's α coefficient for this scale was 0.907. The results of the construct validity analysis (single-factor structure, factor loadings 0.63–0.87, CR = 0.93, AVE = 0.54; acceptance criteria: factor loadings ≥ 0.50, CR ≥ 0.70, AVE ≥ 0.50).

### Peer relationship scale

Peer relationships were assessed using the Peer Relationships Scale developed by Asher and revised by Zhang Yali (Zhang, 2008). The scale consists of three dimensions—acceptance (e.g., “I have many friends”), rejection (e.g., “I can't find anyone to talk to”), and loneliness (e.g., “I feel lonely”)—and includes a total of 16 items, with the following items scored in reverse: 2, 4, 6, 8, 9, 11, 12, 13, 14, and 16. The scale uses a 4-point Likert scale, with scores ranging from 1 to 4 to indicate responses from “strongly disagree” to “strongly agree.” For the reverse-scored items, the raw scores are converted (1 → 4, 2 → 3, 3 → 2,4 → 1). After scoring each dimension separately, the scores for each item across the three dimensions are summed and divided by the number of items to obtain the average score; a higher score indicates better peer relationships. In this study, the Cronbach's α coefficient for this scale was 0.951. The results of the construct validity analysis (single-factor structure, factor loadings 0.60–0.83, CR = 0.91, AVE = 0.52; acceptance criteria: factor loadings ≥ 0.50, CR ≥ 0.70, AVE ≥ 0.50).

### Self-Efficacy Scale

The General Self-Efficacy Scale developed by Schwarzer and revised by Chinese scholars including Wang Caikang (Wang et al., 2001) was used. This scale consists of a single dimension (e.g., “I can handle most problems that arise in my life”) and contains 10 items. It uses a 4-point Likert scale ranging from 1 (strongly disagree) to 4 (strongly agree). The average score is calculated by summing the scores for each item and dividing by the number of items; a higher score indicates greater self-efficacy. In this study, the Cronbach's α coefficient for this scale was 0.924. The results of the construct validity analysis (single-factor structure, factor loadings 0.65–0.89, CR = 0.88, AVE = 0.57; acceptance criteria: factor loadings ≥ 0.50, CR ≥ 0.70, AVE ≥ 0.50).

### Data analysis

Valid data were imported into SPSS 27.0 software for analysis. Pearson correlation analysis, regression analysis, and chained mediation analysis were employed. For the chained mediation analysis, the Process plugin using the Bootstrap method was used to test for mediation effects. When analyzing the indirect effects of variables using the Process plugin, the specific settings were as follows: Model 6 was selected; X represented physical exercise, M1 represented peer relationships, M2 represented self-efficacy, and Y represented social-emotional competence; the number of Bootstrap samples was set to 5,000.

## Results and analysis

### Test for common method bias

Due to factors such as the measurement environment, data obtained from questionnaire surveys may be subject to common-method bias (Zhou and Long, 2004). During the survey implementation phase, procedural measures—including strictly anonymous administration, clarifying that data would be used solely for academic research, emphasizing the voluntary nature of participation and confidentiality, randomizing the order of items across scales, and standardizing the instructions—were employed to minimize participants' fixed response tendencies and subjective concealment biases as much as possible, thereby reducing common-method bias at the source. After data collection, a Harman one-factor test was conducted on the obtained data. SPSS 26.0 was used to analyze physical exercise, peer relationships, self-efficacy, and socio-emotional competence. The results indicated that there were eight factors with eigenvalues greater than 1, and the first factor explained 24.59% of the variance, which was significantly less than the 40% threshold. It should be noted that the Harman one-factor test serves only as a preliminary screening tool for common-method bias and has certain limitations. These results do not indicate the presence of severe common-method bias, but they cannot completely rule out the potential influence of method effects. Based on a comprehensive assessment of the ex ante multiple procedural controls and the ex post preliminary results of the Harman one-factor test, the risk of common-method bias in this study's data falls within an acceptable range and is sufficient to support subsequent mediation model analysis.

### Descriptive statistics for each variable

First, we conducted descriptive statistics for the four core variables—physical exercise, peer relationships, self-efficacy, and social-emotional competence—and performed one-way analysis of variance (ANOVA) grouped by four demographic variables: gender, place of origin, whether the student is an only child, and grade level. The results are shown in Table 2. The mean scores for all variables fell within the upper-middle range of the scale's scoring intervals, with no extreme outliers, thus meeting the data prerequisites for subsequent correlation analysis and mediation modeling. Regarding group differences, gender, whether a child is an only child, grade level, and place of origin did not produce significant between-group differences for either social-emotional competence or self-efficacy; the corresponding η^2^ values were all less than 0.01. According to Cohen's effect size criteria, these findings indicate no practical effect. Regarding physical exercise indicators, significant between-group differences were found for place of origin, whether the student was an only child, and grade level. Among these, the η^2^ values for place of origin and grade level ranged from 0.01 to 0.06, indicating small effects; although the effect of being an only child was statistically significant, η^2^ <0.01, indicating no practical effect. Peer relationships showed significant differences only within the region of origin group, also exhibiting a small effect. These results indicate that individual differences in college students' peer relationships, self-efficacy, and social-emotional competence are not primarily determined by demographic factors such as gender, region of origin, only-child status, or grade level. Instead, individual physical exercise behavior is more likely to be the source of these psychological indicators, providing empirical support for subsequent studies in this research that control for demographic variables and explore the chained mediating role of physical exercise.

**Table 2:** Descriptive statistics for each variable and results of tests for differences across demographic groups (*N* = 517).

Variable	M ±SD	Control variables	*F*	*P*	H^2^
Physical exercise	3.95 ± 0.85	Gender	1.127	0.289	0.002
		Place of origin	9.632	0.002^**^	0.018
		Whether an only child	4.128	0.043^*^	0.008
		Grade	2.715	0.045^*^	0.016
Social-emotional competence	3.90 ± 0.49	Gender	0.552	0.458	0.001
		Place of origin	1.204	0.273	0.002
		Whether an only child	0.025	0.875	0.000
		Grade	1.187	0.314	0.007
Peer relationships	2.60 ± 0.72	Gender	2.015	0.156	0.004
		Place of origin	6.871	0.009^**^	0.013
		Whether an only child	0.337	0.562	0.000
		Grade	1.053	0.368	0.006
Self-efficacy	2.85 ± 0.66	Gender	1.693	0.194	0.003
		Place of origin	1.986	0.159	0.004
		Whether an only child	0.792	0.374	0.002
		Grade	0.881	0.451	0.005

^*^Indicates *P* < 0.05, ^**^indicates *P* < 0.01, and the correlation is significant.

### Correlation analysis of variables

Among the demographic variables, gender, place of origin, and only-child status were dichotomous variables, while grade level was an ordinal variable. Since the distribution of grade levels across the sample was balanced and approximately equidistant, it was treated as a continuous variable. Given that the two-way correlation for dichotomous variables is equivalent to the Pearson correlation, and that this approach is supported by a large body of relevant literature, Pearson correlation analysis was uniformly used to examine the associations between demographic variables and scale scores. Correlation analysis was conducted using the mean scores of each variable. The results revealed that gender was significantly negatively correlated with being an only child and peer relationships; physical exercise was significantly positively correlated with grade level and being an only child, and negatively correlated with place of origin; physical exercise, social-emotional competence, peer relationships, and self-efficacy were significantly positively correlated with one another; no significant correlations were found among the remaining variables (Table 3).

**Table 3:** Correlation analysis of each variables.

Variable	1	2	3	4	5	6	7	8
Gender (1)	1							
Place of Origin (2)	−0.038	1						
Whether an only child (3)	−0.088^*^	−0.226^**^	1					
Grade (4)	−0.076	−0.074	0.058	1				
Physical exercise (5)	0.004	−0.130^**^	0.092^*^	0.093^*^	1			
Social-emotional competence (6)	−0.018	−0.053	0.006	0.053	0.414^**^	1		
Peer relationships (7)	0.066	−0.108^*^	0.011	0.017	0.344^**^	0.350^**^	1	
Self-efficacy (8)	0.046	−0.017	−0.030	−0.028	0.310^**^	0.287^**^	0.306^**^	1

^*^Indicates *P* < 0.05, ^**^ indicates *P* < 0.01, and the correlation is significant.

Gender: 1, Male; 2, Female; Grade Level: 1, Freshman; 2, Sophomore; 3, Junior; 4, Senior; Place of Origin: 1, Urban; 2, Rural; Only Child: 1, Yes; 2, No.

### Test for mediating effects

With physical exercise as the independent variable, social-emotional competence as the dependent variable, and peer relationships and self-efficacy as mediating variables, we used SPSS 26.0 and the PROCESS 3.4 plugin to test the chained mediation effects of peer relationships and self-efficacy on the relationship between physical exercise and social-emotional competence, while controlling for demographic variables such as gender. Model outputs uniformly reported path prediction strengths using standardized regression coefficients; the results are shown in Table 4. The specific steps were as follows: First, with social-emotional competence as the dependent variable, we included gender, grade level, place of origin, and whether the student was an only child as independent variables to control for the potential effects of these variables on college students' social-emotional competence. Second, physical exercise was included to establish a regression model, examining its total effect on social-emotional competence after controlling for the aforementioned variables. The analysis revealed a significant positive correlation between physical exercise and social-emotional competence (β = 0.238, *P* < 0.001), indicating that research hypothesis H1 was supported. Third, the variables of peer relationships and self-efficacy were added to the model to test whether they acted as mediators between physical exercise and social-emotional competence and whether a chained mediation effect existed. The results showed that physical exercise was significantly positively correlated with social-emotional competence (β = 0.173, *P* < 0.001), peer relationships were significantly positively correlated with social-emotional competence (β = 0.141, *P* < 0.001), and self-efficacy was significantly positively correlated with social-emotional competence (β = 0.096, *P* < 0.001). At the same time, the analysis also revealed that physical exercise was significantly positively correlated with peer relationships (β = 0.285, *P* < 0.001) and self-efficacy (β = 0.189, *P* < 0.001), and that peer relationships were significantly positively correlated with self-efficacy (β = 0.205, *P* < 0.001). As can be seen from the above, peer relationships and self-efficacy exert a chained mediating effect between physical exercise and social-emotional competence, confirming research hypotheses H2 and H3.

**Table 4:** Regression model of the mediating effects of peer relationships and self-efficacy.

Regression equation		Overall fit index			Regression coefficient significance	
Dependent variable	Independent variables	*R*	*R^2^*	*F*	β	*t*
Social-emotional competence	Physical exercise	0.416	0.173	21.384	0.238	10.172^***^
Peer relationships	Physical exercise	0.356	0.127	14.888	0.285	8.071^***^
Self-efficacy	Physical exercise	0.384	0.147	14.737	0.189	5.559^***^
	Peer relationships				0.205	5.128^***^
Social-emotional competence	Physical exercise	0.486	0.237	22.592	0.173	7.044^***^
	Peer relationships				0.141	4.910^***^
	Self-efficacy				0.096	3.117^**^

^**^Indicates *P* < 0.01.

^***^Indicates *P* < 0.001.

Using the Bootstrap method with 5,000 repeated samples, we tested the mediating effects of peer relationships and self-efficacy on the relationship between physical exercise and social-emotional competence, and the results of the confidence intervals are shown in Table 5. The total effect of physical exercise on social-emotional competence was 0.238, and its 95% Bootstrap confidence interval did not include 0 (LLCL = 0.192, ULCL = 0.284), indicating that both the total effect of physical exercise on social-emotional competence and the mediating effects of the two variables were significant. The direct effect of physical exercise on social-emotional competence was 0.173 (direct path), and the Bootstrap 95% confidence interval did not include 0 (LLCL = 0.125, ULCL = 0.222), indicating that physical exercise has a significant direct effect on social-emotional competence, accounting for 72.69% of the total effect.

**Table 5:** Mediating effects of peer relationships and self-efficacy.

Effect	Path	Effect size	Standard error	LLCL	ULCL	Effect ratio
Total effect		0.238	0.023	0.192	0.284	100%
Direct effect	Direct path	0.173	0.025	0.125	0.222	72.69%
Total indirect effect		0.065	0.011	0.044	0.088	27.31%
	Path 1	0.041	0.009	0.023	0.060	17.23%
Indirect effects	Path 2	0.018	0.007	0.007	0.032	7.56%
	Path 3	0.006	0.002	0.002	0.011	2.52%

The model shows that physical exercise is positively correlated with social-emotional competence, and that peer relationships and self-efficacy play indirect mediating roles. There are three pathways in total, with a total indirect effect of 0.065. The 95% Bootstrap confidence interval for the total indirect effect does not include 0 (LLCL = 0.044, ULCL = 0.088), accounting for 27.31% of the total effect. Specifically, the first mediation path—physical exercise → peer relationships → social-emotional competence (Path 1)—has an indirect effect of 0.041, accounting for 17.23% of the total effect; the second path: physical exercise → self-efficacy → social-emotional competence (Path 2), with an indirect effect of 0.018, accounting for 7.56% of the total effect; and the third chained mediation path: physical exercise → peer relationships → self-efficacy → social-emotional competence (Path 3), with an indirect effect of 0.006, accounting for 2.52% of the total effect, indicating that research hypothesis H4 is supported.

**Figure 2:**
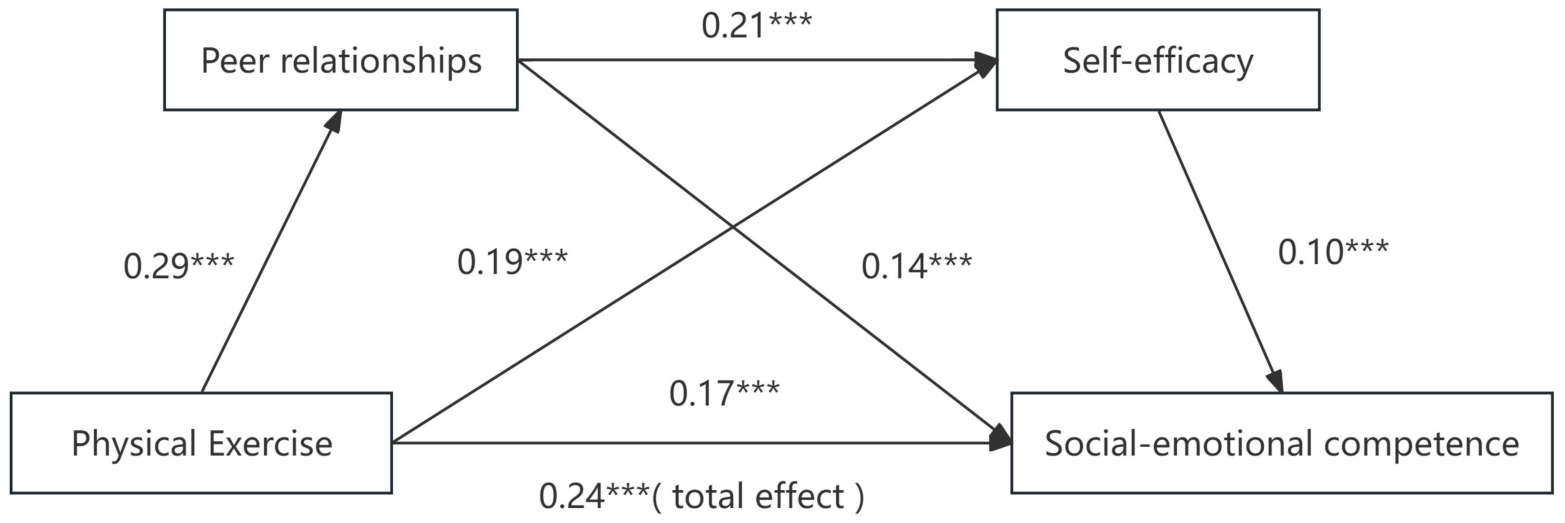
Chain mediation model of peer relationships and self-efficacy.

Based on the above findings, the chained mediation model is shown in Figure 2.

## Discussion

### The relationship between physical exercise and social-emotional competence among college students

This study found a significant positive correlation between physical exercise and social-emotional competence among college students. Specifically, students who engage in higher levels of physical exercise tend to exhibit higher social-emotional competence, thereby confirming Hypothesis 1. As a positive behavioral intervention, physical exercise is closely linked to an individual's holistic development and serves as a key factor in fostering social-emotional competence among college students (Goh et al., 2022). Chen Zhaojun argues that college students' social-emotional competencies encompass dimensions such as personal relationship skills, interpersonal communication skills, group relationship skills, and responsibility (Chen et al., 2023). First, personal relationship skills refer to an individual's ability in areas such as self-awareness and emotional regulation; physical exercise can cultivate college students' self-awareness and emotional regulation abilities. During physical exercise, students must constantly monitor their physical condition, a process that deepens their self-awareness (Thapa et al., 2024). Based on previous research, this study hypothesizes that exercise triggers the secretion of neurotransmitters such as endorphins and dopamine, which may enable individuals to maintain emotional stability by overcoming negative emotions, thereby improving their emotional regulation abilities (Peng et al., 2025). Second, the interactions that occur during physical exercise provide college students with opportunities for interpersonal engagement. Whether through collaboration in team projects or competitive matches, students can adjust their behavior through verbal and nonverbal communication. This process may enhance students' collaboration and interpersonal skills (Du and Liu, 2022). Third, China's collectivist culture emphasizes synergy and cooperation within organizations; team sports in physical education reinforce students' sense of belonging and cultivate their ability to function as part of a collective in a complex society (Phipps et al., 2015). Finally, the rules and strategies of competitive sports cultivate students' sense of responsibility; students must weigh consequences, make decisions that comply with rules and ethical standards, and take responsibility for their actions (Liu et al., 2021). This sense of responsibility developed through sports can be effectively transferred to academic and daily life. In summary, there is a significant positive correlation between physical exercise and college students' social-emotional competencies.

### The mediating role of peer relationships

Research confirms that peer relationships play a significant mediating role between physical exercise and social-emotional competencies, thereby validating Hypothesis 2. First, we found a positive correlation between physical exercise and peer relationships. Participating in physical exercise provides college students with opportunities to broaden their interpersonal interactions and deepen the acceptance and recognition within their social circles, thereby improving their peer relationships (Ma et al., 2022). When college students engage in physical exercise with their peers, this support and collaboration promote harmonious coexistence within the group. The emotional and social support provided through these physical activities not only generates positive and enjoyable experiences but also fosters a sense of belonging among students. Second, we found a positive correlation between peer relationships and social-emotional competence. Sullivan's theory of interpersonal relationships posits that peer relationships are one of the factors in an individual's socialization and the formation of a healthy personality, playing a crucial role in the development of self-awareness and interpersonal skills (Sullivan, 2013). Individuals with positive peer relationships demonstrate particularly high levels of performance in dimensions such as emotional understanding, cognitive abilities related to emotional expression, and the acceptance of others' emotional perspectives (Lan and Wang, 2019). More importantly, peer relationships exert a strong positive influence on emotional regulation, enabling individuals to engage in more adaptive emotional regulation and fostering the development of social-emotional competencies (Deng et al., 2023). Given that Chinese college students tend to express emotions in a reserved manner and exhibit stable social behavior, their social-emotional competencies may be related to a positive peer environment (Wu, 2023). Therefore, peer relationships exert a separate mediating effect between physical exercise and college students' social-emotional competencies.

### The mediating role of self-efficacy

Research has confirmed that self-efficacy plays a positive mediating role between physical exercise and social-emotional competence, thereby validating Hypothesis 3. A study by N. Gutiérrez Ángel et al. on a group of college students found a positive correlation between physical exercise and self-efficacy (Mercader-Rubio et al., 2023). Regular physical exercise can provide individuals with psychological benefits such as a sense of accomplishment, psychological satisfaction, self-efficacy, and positive emotional changes (Mutz et al., 2021). At the same time, the level of physical exercise is positively associated with the development of an individual's confidence and psychological resilience in coping with social challenges (Doorley et al., 2022). Furthermore, we found that self-efficacy is positively correlated with social-emotional competence. Research has shown that students with higher self-efficacy are more likely to leverage positive interpersonal relationships to enhance their emotional regulation, interpersonal skills, and overall social competence (Eriksen and Bru, 2023). Students with higher self-efficacy typically exhibit greater emotional resilience and more adaptive coping strategies; these psychological assets can promote the development of their social-emotional competence (Baños et al., 2023). Furthermore, individuals with higher levels of self-efficacy are generally more receptive to emotional input and are more likely to exhibit emotionally intelligent behaviors in interpersonal interactions (Hoyt and Blascovich, 2010). Therefore, self-efficacy plays a separate mediating role between physical exercise and college students' social-emotional competencies.

### Chain mediation of peer relationships and self-efficacy

This study examined the interrelationships among physical exercise, college students' social-emotional competencies, peer relationships, and self-efficacy, revealing that peer relationships and self-efficacy not only mediate the relationship between physical exercise and social-emotional competencies independently but also exert a chained mediating effect. Specifically, a positive correlation exists between peer relationships and self-efficacy, thereby confirming Hypothesis 4. Physical exercise serves as an important means of strengthening peer relationships among college students; regular physical exercise enables individuals to discover shared interests with their peers, enhances interpersonal relationships, and facilitates the establishment of positive peer relationships (Fitzgerald et al., 2012). Research indicates that peer relationships are positively correlated with self-efficacy. For example, vicarious success experiences among peers can generate strong psychological cues, leading individuals to believe they also possess the ability to complete tasks, thereby fostering a sense of self-efficacy (Kudo and Mori, 2015). Positive evaluations and social persuasion from peers can effectively alleviate an individual's sense of uncertainty when facing new environments or high-pressure tasks, reduce anxiety about failure, and encourage individuals to evaluate their own potential more positively (Nob, 2021). More notably, unlike Western individualism, which emphasizes the independent self, traditional Chinese culture places greater importance on collective belonging [Pan (2023)]. Influenced by collectivist culture, college students tend to rely on peer groups for self-identity and place greater importance on teamwork and group dynamics; peer relationships play a more prominent role in shaping their self-efficacy (Ma et al., 2023). Furthermore, self-efficacy is positively correlated with social-emotional competence. Based on previous research, this study hypothesizes that a strong sense of self-efficacy may enhance the prefrontal cortex's regulatory function over the limbic system, enabling individuals to employ positive emotional regulation strategies with greater confidence (Li and Liu, 2025), thereby helping college students achieve better developmental outcomes in areas such as personal relationships, social interactions, group collaboration, and responsibility-taking, which are closely linked to the development of their social-emotional competencies. Thus, peer relationships and self-efficacy exert a chained mediating effect on the relationship between physical exercise and college students' social-emotional competencies.

## Limitations and future directions

This study explores the relationship between physical exercise and college students' social-emotional competencies. Theoretically, it expands the research field on the relationship between physical exercise and college students' social-emotional competencies, clarifies the chained mediating role of peer relationships and self-efficacy in this relationship, and provides a new perspective for understanding the factors related to college students' social-emotional competencies. In practice, the findings of this study provide practical guidance for fostering college students' social-emotional competencies. Universities and society should place high importance on the positive role of physical exercise in shaping students' positive behavior. By formulating incentive policies and offering diverse physical exercise programs, they can foster a positive atmosphere that encourages active participation in physical exercise and effectively improve students' levels of physical exercise. In line with China's “Five-Education Approach” and the routine integration of mental health education, universities can leverage multidimensional pathways—including physical education classes, extracurricular physical exercise, and campus sports culture—to harness the social educational value of physical exercise. For example, universities can optimize their public physical education curriculum by adding team-building activities, team ball sports, and group outdoor adventure sports, using group sports settings to help students build healthy peer relationships. University student affairs offices, counseling centers, and athletics departments can collaborate on joint initiatives to integrate physical exercise into the mental health education intervention system for college students, regularly organizing distinctive activities such as stress-relief through exercise and fun group competitions.

However, this study also has some limitations. First, the study employed a questionnaire survey method, and the data is based on self-reports from college students, which may involve some subjective bias. Self-reported data can only reflect correlations between variables; they cannot determine the direction of causality, leaving open the possibility of reverse causality: college students with higher social-emotional competencies may also be more proactive in participating in physical exercise, more likely to establish positive peer relationships, and perceive higher levels of self-efficacy, suggesting that there may be alternative explanatory pathways for the relationships among these variables. Additionally, this study relied solely on Harman's one-way test for preliminary screening of common method bias, a method that has its limitations. Future research may employ more robust procedural or statistical methods, such as multi-source data, temporal separation, marker variables, or latent method factors. Second, the study sample was limited to college students in Liaoning Province, which introduces certain regional limitations. Significant differences exist across regions in terms of economic development, regional culture, and educational resources; the regional development characteristics of Liaoning Province cannot be generalized to other regions of China, such as the western and southern parts of the country. Furthermore, this study is cross-sectional in nature; future research could adopt longitudinal or experimental designs to further validate the causal relationships among physical exercise, peer relationships, self-efficacy, and social-emotional competence. Finally, this study only examined the mediating roles of peer relationships and self-efficacy in the relationship between physical exercise and college students' social-emotional competencies; other mediating variables, such as social support and school climate, may also exist. Therefore, future research will incorporate multiple variables to systematically explore the interaction mechanisms among these variables.

## Conclusion

1) Physical exercise, peer relationships, and self-efficacy are all positively correlated with each other and with college students' social-emotional competencies.

2) Physical exercise is positively correlated with college students' peer relationships, self-efficacy, and social-emotional competence; peer relationships are positively correlated with college students' self-efficacy and social-emotional competence; and self-efficacy is positively correlated with college students' social-emotional competence.

3) Peer relationships and self-efficacy each play an independent mediating role between physical exercise and college students' social-emotional competencies.

4) Peer relationships and self-efficacy exert a chained mediating effect on the relationship between physical exercise and college students' social-emotional competencies.

## Data Availability

The original contributions presented in the study are included in the article/supplementary material, further inquiries can be directed to the corresponding author.
